# Effects of the *Escherichia coli* Bacterial Toxin Cytotoxic Necrotizing Factor 1 on Different Human and Animal Cells: A Systematic Review

**DOI:** 10.3390/ijms222212610

**Published:** 2021-11-22

**Authors:** Francesca Carlini, Zaira Maroccia, Carla Fiorentini, Sara Travaglione, Alessia Fabbri

**Affiliations:** 1 Department of Cardiovascular, Endocrine-Metabolic Diseases and Ageing, Istituto Superiore di Sanità, 00161 Rome, Italy; francesca.carlini@iss.it (F.C.); zaira.maroccia@iss.it (Z.M.); sara.travaglione@iss.it (S.T.); 2 Associazione Ricerca Terapie Oncologiche Integrate, ARTOI, 00165 Rome, Italy; carla.fiorentini@artoi.it

**Keywords:** Rho GTP-binding proteins, cytotoxic necrotizing factor type 1, actin cytoskeleton, mitochondria, apoptosis, primary cell culture, transformed cell line, cancer cell line

## Abstract

Cytotoxic necrotizing factor 1 (CNF1) is a bacterial virulence factor, the target of which is represented by Rho GTPases, small proteins involved in a huge number of crucial cellular processes. CNF1, due to its ability to modulate the activity of Rho GTPases, represents a widely used tool to unravel the role played by these regulatory proteins in different biological processes. In this review, we summarized the data available in the scientific literature concerning the observed in vitro effects induced by CNF1. An article search was performed on electronic bibliographic resources. Screenings were performed of titles, abstracts, and full-texts according to PRISMA guidelines, whereas eligibility criteria were defined for in vitro studies. We identified a total of 299 records by electronic article search and included 76 original peer-reviewed scientific articles reporting morphological or biochemical modifications induced in vitro by soluble CNF1, either recombinant or from pathogenic *Escherichia coli* extracts highly purified with chromatographic methods. Most of the described CNF1-induced effects on cultured cells are ascribable to the modulating activity of the toxin on Rho GTPases and the consequent effects on actin cytoskeleton organization. All in all, the present review could be a prospectus about the CNF1-induced effects on cultured cells reported so far.

## 1. Introduction

Cytotoxic necrotizing factor 1 (CNF1) is a bacterial virulence factor associated with some pathogenic *Escherichia coli* strains causing urinary tract infection and meningitis [1]. It belongs to the cytotoxic necrotizing factors family that includes proteins from *E. coli* (CNF1, CNF2, and CNF3) and *Yersinia pseudotuberculosis* (CNFY). CNF1 is an AB-type toxin, composed of a cell-binding domain and the C-terminal catalytic domain, bearing deamidase activity. The cell-binding domain encompasses two interaction sites in CNF1: an N-terminus domain, which interacts with the 37-kDa laminin receptor precursor (LRP), and a domain directly adjacent to the catalytic domain, which is a high affinity interaction site for the Lutheran (Lu) adhesion glycoprotein/basal cell adhesion molecule (BCAM) Lu/BCAM [2,3]. Following endocytosis, the catalytic domain of CNF1 is cleaved off and released into the cytosol [4]. The CNF1 target is represented by small GTPases belonging to the Rho family, Rho, Rac, and Cdc42. CNF1 deamidates a specific glutamine residue, located in the switch 2 domain and involved in GTP hydrolysis (glutamine 63 in RhoA [3,5,6] or 61 in Cdc42 and Rac1 [7]) and this modification results in the constitutive association of the Rho GTPase with GTP, namely, its constitutive activation.

Nonetheless, some of the Rho-regulated signaling pathways have been found to be only transiently activated. That is because, once constitutively activated by CNF1, Rho proteins are rapidly conveyed to the ubiquitin-mediated proteasomal degradation pathway [8,9]. Interestingly, degradation seems to be cell type-specific. For example, while in HUVECs, macrophages, keratinocytes, fibroblasts, and 804G cells, the three CNF1-activated GTPases undergo efficient ubiquitin-mediated proteasomal degradation, in HEp-2, Vero, and HEK293 cells the ubiquitination level of specific Rho proteins is quite low. In these cell lines, a specific absence of cellular depletion of Rho (Vero), Cdc42 (HEK293), and Rac (HEp-2) is shown, indicating the existence of three independent and differently expressed ubiquitination pathways for the three GTPases [8,10,11].

Rho GTPases are signaling nodes, regulated by diverse upstream extracellular stimuli, and interacting with a wide range of downstream effectors that initiate a number of cellular signaling cascades. Rho GTPases are mainly known for their role in the modulation of cytoskeletal dynamics and, as a consequence, of cell adhesion, migration, and endocytosis [12,13]. Rho controls actin stress fiber formation and contractile actomyosin bundles found in many cultured non-muscle cells and plays a central role in cell adhesion and morphogenesis. Rac regulates the formation of membrane ruffles, while Cdc42 is involved in the formation of filopodial extensions at the leading edge, both characteristic features of many actively migrating cells.

Beyond the involvement in direct regulation of the actin cytoskeleton, Rho GTPases play a key role in a huge number of crucial cellular processes, such as the regulation of transcription, cell polarity, cell cycle progression and inflammation [10]. They are also involved in physiological processes, including embryonic development [14], neuronal differentiation and neurite formation [15,16], maintenance of stem cells [17,18], and both innate and adaptive immune cell processes [19].

Hence, due to its ability to modulate the activity of Rho GTPases, CNF1 represents a widely used tool to unravel the role played by these regulatory proteins in several biological processes [20].

From a bacterial point of view, Rho GTPases, by activating a panel of effectors, confer to pathogens the ability to alter the architecture of host cells and tissues, thereby promoting their ability to evade host defenses and spread within and among hosts [21]. In this context, CNF1 has been investigated as a potential risk factor for cancer onset and/or progression, especially in those anatomical areas that naturally host *E. coli* pathogenic strains (colon, uroepithelial tract) [22,23].

The aim of this systematic review is to summarize the data available in the scientific literature concerning the observed in vitro effects induced by CNF1 in different cell lines of finite/primary, transformed/immortalized, and tumoral origin.

## 2. Materials and Methods

### 2.1. Literature Search Strategy

The search was oriented on original peer-reviewed scientific articles in which any CNF1-induced effect observed in in vitro studies was reported.

According to the Preferred Reporting Items for Systematic Reviews and Meta-Analyses (PRISMA) guidelines [24], a systematic review of the published literature from inception to 30 June 2021 was performed in electronic bibliographic databases (US National Library of Medicine MEDLINE/PubMed, EMBASE, Web of Science, and Scopus). An internet-based search was also performed. The electronic search was conducted by three authors independently (F.C., Z.M., and A.F.) using a combination of keywords (“CNF1”, “primary cells”, “transformed cells”), according to the different search instructions and techniques adopted in each database (Figure S1).

Selected publications were compiled into a single database and duplicates removed.

Based on the inclusion/exclusion criteria, two authors independently (F.C. and S.T.) screened all the publication records identified and reviewed the abstracts to identify articles requiring an additional full-text review. The final decision was reached through consensus, solving discrepancies by discussion with a third author (A.F.).

Full-texts of the selected publications were obtained. The reference lists of the selected articles were also checked, in order to identify further works eligible for this study.

### 2.2. Inclusion Criteria

For this review, only original peer-reviewed scientific articles reporting morphological or biochemical modifications induced in vitro by soluble CNF1 (recombinant or from pathogenic *E. coli* extracts highly purified with chromatographic methods) were selected. Results related to both human and animal cells were included. Only English-written studies were included.

### 2.3. Exclusion Criteria

Irrelevant articles, letters to the editor, editorials, case reports, reviews, short communications, bioinformatic meta-analyses, and articles written in languages other than English were excluded. Papers focused on in vivo studies, on receptor studies or those in which pathogenic *E. coli* strains were used for infection, were not included, as well as CNF1-induced effects reported as “data not shown”.

### 2.4. Data Extraction

Two authors (F.C. and S.T., independently) screened the abstracts and the full-text versions of the selected articles to double-check their eligibility and achieve data extraction. The selected articles were further verified by two authors (C.F. and A.F.). Of the 239 articles examined, 76 met the eligibility criteria fixed in the present review.

The following information was obtained from each paper: cell line name, cell line, tissue origin, bibliographic references, summary of the observed effects ascribed to the toxin, and the type of CNF1 preparation used.

All eligible studies were grouped according to the cell line in which CNF1 effects have been examined: cancer, immortalized/transformed, and finite/primary.

## 3. Results

Using combinations of the keywords “CNF1”, “primary cells”, and “transformed cells” (Figure S1), a total of 299 studies were identified through literature research in the search engine, of which 75 were removed as duplicates. Fifteen records were identified from other sources. After screening the abstracts and full-text, 163 studies were excluded. The remaining 76 papers were finally assessed for eligibility.

**Figure 1:**
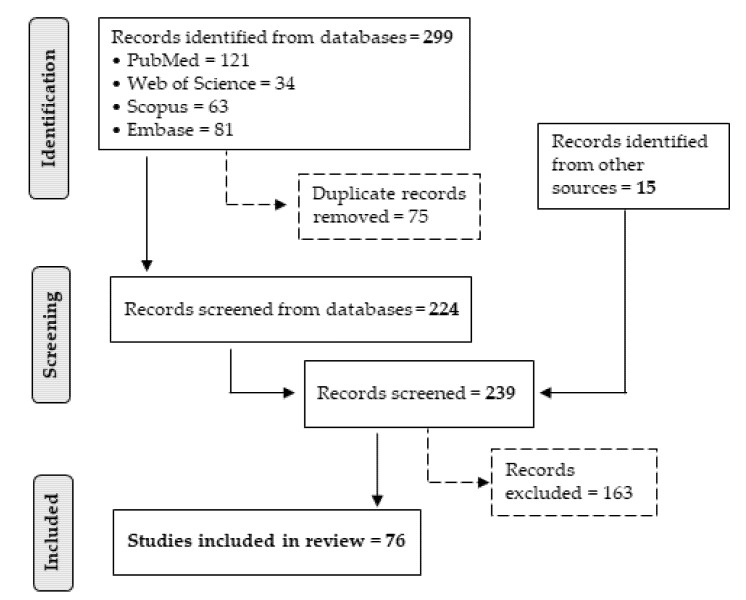
PRISMA flow diagram. Flowchart of the selection process for the inclusion of studies.

The flow diagram in Figure 1 summarizes the selection process, showing the number of records passing through each step.

The search identified 76 experimental studies focused on 74 cell lines.

In Table 1, Table 2 and Table 3, the observed effects induced by CNF1 toxin and the related references have been grouped together for each cell type origin i.e., cancer, immortalized/transformed, and finite/primary.

### 3.1. Effects on Rho GTPases and on Actin Cytoskeleton

Not all selected papers report the description of the CNF1 effects on its direct targets, Rho, Rac, and Cdc42 (i.e., analyzed by pulldown, band shift, or glutamine 63 deamidation experiments), probably depending on the specific purpose of the single experimental work.

Overall, the specific activity of CNF1 is to permanently activate Rho, Rac, and Cdc42 GTPases in all the cell systems where its effects have been studied [2,4,5,7,8,9,10,25,26,27,32,33,34,35,36,37,38,39,41,42,43,47,52,55,56,57,58,59,60,61,62,63,64,68,69,75,77,78,79,80,81,84,89,90,91,92]. However, the timing and level of activation of the distinct members of the Rho GTPase family may turn out to be variable between different cell types and lines. It is known that Rho proteins and/or their regulators (GDIs, GAPs and GEFs) can be differentially expressed, due to the specific phenotype and/or physiological conditions of a specific cell line [93]. Moreover, of great importance is the efficiency of the ubiquitination/proteasomal degradation system of the host cell. Indeed, CNF1-activated Rho GTPases undergo polyubiquitination, a modification targeting Rho proteins to the degradative proteasome machinery, a crucial mechanism in the control of both Rho small GTPases and their modulators. The observed Rho GTPase depletion after CNF1 treatment is strictly dependent on its sustained activation [8,9,11,33,37,59,94,95].

**Figure 2:**
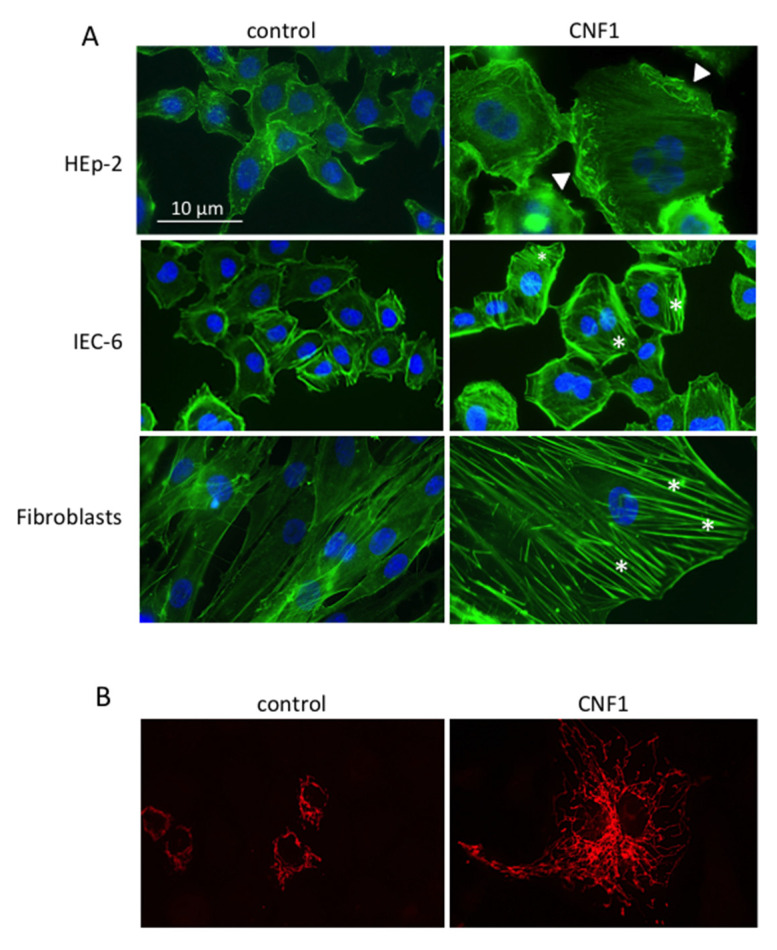
Example of morphological effects of CNF1 on actin and mitochondria. (**A**) F-actin and nuclei staining of different cell lines untreated or treated with CNF1. Asterisks: stress fibers; arrow-heads: ruffles. (**B**) Mitochondrial staining of control and CNF1-treated IEC-6 cells. Note the enrichment of the mitochondrial network in treated cells.

In most studies, the induction of at least some of the morphological effects characteristic of Rho GTPase activation is described, that is, changes in the actin cytoskeleton organization, demonstrated by actin stress fibers or the formation of actin cables, membrane ruffles, filopodia and lamellipodia assembly (Figure 2A) [6,7,8,10,25,26,32,34,35,36,38,40,41,44,45,46,47,50,51,52,56,94,95].

#### 3.1.1. Actin Cytoskeleton-Dependent Phenomena (Motility, Focal Adhesion, Permeability, Phago-Pinocytosis)

Regardless of the method used to identify the impact of CNF1-induced cytoskeletal modifications on cell movement ability (migration—invasion test, scratch wound healing assay), CNF1 treatment stimulated an increase of cell motility in 13 cell lines (T24, 5637, HUVEC [28]; HT-29, SW480, HEp-2 [22]; PC3, LNCaP, 22Rv1, VCaP [62]; BL6-10 [64]; 804G, HUVEC [8]; T-lymphocytes [44]). Among these, in PC3 [62] and BL6-10 [64] cells, this effect was accompanied by an in vivo increase in metastatic ability.

By contrast, in five cell lines, the toxin showed an inhibitory effect on cell motility (T84 [43]; HeLa [36,38]; GL216 [32]; HPECC, HBEC-5i [22]).

Of interest, in T-lymphocytes and IEC-6 cells, CNF1 was able to raise cell motility, but only in the presence of SDF-1α [44] or inflammation mediators [22].

In four CNF1-treated cell lines, an increase in focal adhesion formation was observed, irrespective of whether cell motility was increased or inhibited (BL6-10 [64,65]; Caco-2 [39]; HeLa [36]; IGR-Heu-R8 [59]).

Effects of CNF1 treatment on phagocytic activity has been observed. In particular, two publications [60,95] report that, in different monocyte/macrophagic cell lines, of both primary and cancer origin (BMDM, mouse peritoneal macrophages, human monocytes, THP-1, Raw264.7), CNF1 reduced the phagocytosis of nonopsonized beads and of nonopsonized bacteria. Conversely, in HEp-2 and in 804G epithelial cancer cell lines, CNF1 confers the ability to ingest latex beads as well as bacteria [8,46,47,54].

These results suggest that CNF1 might contribute to bacterial infection by favoring epithelium colonization and/or affecting the host innate immune defense, thus reducing the pathogenic *E. coli* clearance ability of macrophages (by decreasing scavenger receptor CD36 expression).

Along with cytoskeletal modifications, alterations in the distribution or in the amount of intercellular junction proteins following CNF1 treatment are described in eight cells lines (HT-29, IEC-6 [22]; T84 [42]; MyEnd [71,72]; HMEC1 [67]; MDCK [77]; HDMEC, PAEC [70,79]). For example, E-cadherin, β-catenin, zonula occludens-1 (ZO-1), caveolin-1, as well as junction adhesion molecule-1 were reorganized away from the TJ membrane (MyEnd [71]; MDCK [77]; HT-29 [22]). In some cases, cytoskeletal and tight junction alterations were accompanied by modifications in cell monolayer permeability, as in two colon carcinoma cell lines in which CNF1 caused a transient rise in cell monolayer paracellular permeability (Caco-2 [39,40]; T84 [41,42,54]) and the transepithelial migration of polymorphonuclear neutrophils (PMN) (T84 [41,42,54]).

On cell lines of endothelial origin, activation of Rho GTPases by CNF1 seems to have different effects on the regulation of cell permeability depending on the background of the endothelial cell lines. Baumer and co-workers [70] show, in fact, that CNF1-induced activation of Rho GTPases reduces permeability in microvascular endothelial cell types; whereas, in macrovascular endothelial cells CNF1 stabilizes barrier functions.

#### 3.1.2. Multinucleation, Cell Cycle, Cell Death, Apoptosis, and Senescence

It is well known that Rho signaling pathways are involved in cell proliferation and cell cycle regulation, also through actin cytoskeleton regulation.

Multinucleation after treatment with CNF1 is reported for 19 cell lines (HEp-2 [27,49,50,53,57,96]; HeLa [33,34,36]; Caco-2 [39]; HT-29 [22]; T24 [26,27]; 5637 [26,27]; Y-1 [27]; A-498 [27]; J82 [27]; SV-HUC-1 [27]; ACHN [27]; human GMB [32]; GL261 [32]; JURKAT [44]; PAE [78]; NIH 3T3 [6,37]; 3T3-Swiss Albino [53]; 3T3-L1 [75]; HCT-116 [45]) (Figure 2A). These morphological changes are probably a consequence of CNF1-induced mitosis/cytokinesis failure [1,49,97]. In fact, it is well known that Rho GTPases are involved in several stages of mitosis, such as spindle formation and attachment to the kinetochore, as well as in the cytokinesis process [98]. Moreover, CNF1 is classified as a cyclomodulin, thus being able to perturb the host cell cycle [99]. Actually, along with multinucleation in some of the reported works, CNF1 treatment is shown to induce a block or a partial inhibition of cell proliferation (GL261 [32]; 3T3-L1 [75]; BL6-10 [64]; Hs 738 [100]) and/or G2/M arrest (T24, 5637 [26]; HEp-2 [50]; HeLa [34]), accompanied by a downregulation of cyclin B1 expression and its cytoplasmatic sequestration (T24 [26]; HEp-2 [50]).

In four cell lines, the inhibition of cell cycle progression leads cells to a senescence state (U87 GL261, human GBM [31]; HCT-116 [45]). Of interest, Zhang and co-workers [45] showed that in HCT-116 human colon cancer cells, CNF1 elicited endoreplication and polyploidization driving cells into a reversible senescence state, which provided a survival route to the cells via depolyploidization. Indeed, authors showed that when CNF1-induced polyploid cells were cultured in fresh medium, in the absence of the toxin, a population of depolyploidized cells able to re-enter the mitotic cycle was selected [45]. Importantly, progeny derived from the CNF1 treatment exhibited genomic instability exemplified by an increased aneuploidy.

In three cell lines, after prolonged treatment, the block of proliferation resulted in cell death (5637 [27]; 3T3-L1 [75]; GL261 [32]), which in one case occurred by an apoptotic mechanism (5637 [27]). In other cell lines, CNF1 treatment seems to protect cells from apoptosis induced by exposure to UV [57,96] or in simulated microgravity conditions [64,65]. Although the molecular mechanisms are still unknown, it seems reasonable, that the fate (senescence, cell death, or survival) of CNF1-treated cells largely depends on the cell type and on the transformation degree of the cells exposed.

### 3.2. Mitochondria and Mitochondria-Related Phenomena

The effect of CNF1 on mitochondrial activity has been analyzed in seven cell lines (BL6-10 [64]; HEp-2 [47,51,58,96]; T24 [25]; SH-SYS5 [29]; IEC-6 [81]; human fibroblasts MERRF [84]). Overall, CNF1 seems to affect mitochondrial metabolism by stimulating an increase in ATP synthesis (IEC-6 [81]; human fibroblasts MERRF [84]) and counteracting the negative effects produced on these organelles under particular experimental or pathological conditions [29,64,84]. In four of the above-mentioned cell lines (SH-SY5Y, HEp-2, IEC-6, human fibroblasts MERRF), metabolic stimulation was accompanied by a prominent modification in the mitochondrial morphology, consisting of the formation of a complex network of elongated organelles (Figure 2B). This was probably due to phosphorylation/inactivation of dynamin-related protein 1 (Drp1), one of the large GTPases that control the mitochondrial fission process.

Indeed, in IEC-6 and SH-SY5Y cells a significant increase in the phospho-Drp1 protein was observed (IEC-6 [81]; SH-SY5Y [29]).

### 3.3. CNF1 on Immune Cells

Few articles investigated CNF1-induced effects in cells of the immune system.

In both finite/primary (human monocytes, macrophages, DC monocytes, mouse peritoneal macrophages) and cancer monocytic/macrophagic cell lines (THP-1, RAW264.7), CNF1 is able to modulate CR3 activation and its colocalization with the actin cytoskeleton (THP-1 e monocytes [60]) and downregulate CD36 transcription/expression [96], leading to a reduced phagocytic ability of nonopsonized beads and/or *E. coli* bacteria (THP-1, RAW264.7, mouse peritoneal macrophages [96]; THP-1, human monocytes [60]).

On the other hand, one paper shows that CNF1 triggers the activation and phenotypic maturation of cultured monocyte-derived DCs, with an increased level of IL-6 and TNF-α secretion and the proliferation of allogenic naïve CD4+ T cells (DC monocytes [83]). In bone-derived macrophages, CNF1 toxin activates the NLRP3 inflammasome via a signaling cascade that involves PAK1/Rac2, thus inducing caspase-1 activation and IL1-β secretion [85].

In cells of lymphoid origin, both primary (T-lymphocytes, BMDM) and leukemic (Jurkat), CNF1 treatment enhances cell migration ability across acellular filters and their adherence to colonic epithelial cell monolayers. In particular, treated T-lymphocytes are able to adhere more tightly to monolayers of human intestinal epithelial cell lines resulting in cytotoxicty for the epithelial cells. In these cells, CNF1 also stimulates the production of high levels of TGF-β1, TGF-β2, TGF-β3, and TNF-α proteins (Jurkat, T-lymphocytes [44]).

In NK cells, CNF1 causes a strong increase in the binding efficiency and killing capacity of effector cells. An augmented expression of cell adhesion and activation-associated molecules, as well as reshaping of the actin and microtubule networks, are also described and probably represent the basis of the enhanced binding ability and cytotoxicity of NK-treated cells [82].

Overall, the in vitro described effects of CNF1 toxin on cells of immune origin suggest its ability to affect innate immune defenses, facilitating bacterial infection and increasing the virulence of *E. coli* pathogenic strains (in the intestinal epithelia). On the other hand, CNF1 also seems to elicit a protective immune mechanism, which is consistent with in vivo studies indicating CNF1 as promoter of antibacterial immunity [101,102]. This apparent discrepancy between pro- and antibacterial activity induced by CNF1 probably depends on the experimental settings and on the specific purpose of the study.

### 3.4. CNF1 Effects on Different Cellular Pathways

Rho proteins have over 60 known downstream effectors, which determine the outcome of activation for a given Rho GTPase protein. The activation of CNF1-induced Rho GTPases affects different cellular pathways that, in turn, drive different new cell states. Actually, in the reviewed papers, CNF1 cell intoxication resulted in a number of proteins being modulated. The described effects on actin organization and cytoskeletal rearrangement (see Section 3.1.1.) are often accompanied by modifications in the distribution and/or expression of proteins involved in specific signal transduction pathways regulating cytoskeleton organization, as well as cell adhesion, motility, and migration.

Both Rho GTPases and cytoskeletal rearrangements are also known to influence gene transcription [93]. Actually, after CNF1 intoxication, in 17 cell lines (HeLa [38]; BL6-10 [65]; HEp-2 [48,52]; GL-261 [31]; C2C12 [76]; HUVEC [10]; 3T3-L1 [75]; mouse peritoneal macrophages, Raw264.1, BMDM, THP-1 [96]; 5637, T24 [28]; HT-29, IEC-6 [22]; 293T [68]) a number of transcription factors (TFs) also result in being modulated. For example, in HEp-2 epithelial cells, CNF1 activates NF-κB through the Rac1/PI3K/Akt/IKK prosurvival pathway, with the ensuing modulation of the antiapoptotic proteins Bcl-2 and Bcl-XL [51,95].

Moreover, CNF1 promotes transcription and release of proinflammatory cytokines, such as IL-6, IL-8 and TNF-α, in cells of different origin (T24 [25]; HEK 293T [68]; HUVEC [10]; human T-lymphocytes [44]) and DC monocytes [83]. Furthermore, CNF1 upregulates the transcription of cyclooxygenase-2 [86], as well as the cell adhesion molecule ICAM-1 (human NK [82]), and the cell cycle related genes p21 and p16 (U87, GL261, human GBM [31,45]). Interestingly, the genes coding for all the above-mentioned proteins are under NF-κB TF control [103,104].

One more example shows how, in both 5637 and T24 uroepithelial cell lines, under hypoxic conditions, CNF1 indirectly promotes VEGF secretion and angiogenesis through RhoC-dependent activation of the HSF1-HSP90α-HIF1α axis. In particular, activated RhoC induces HIF1α stabilization and VEGF production by increasing HSP90α expression and the interaction between HIF1α and HSP90 [28]. Beyond the mentioned examples, other TFs, as well as proteins, are described in the literature as modulated after CNF1 cell treatment (IκB-α, c-Jun [10]; MyoD [76]; C/EBP-α, PPAR-γ [75]; AP-1 [38]; FoxG1 [31]; C/EBP-α, LXR-β [96]; Snail1, ZEB1 [22]).

It is evident that the heterogeneity of the reported results reflects the great variability of the experimental models, cell lines, experimental conditions, and authors’ purpose between the different reviewed articles.

## 4. Discussion and Conclusions

**Figure 3:**
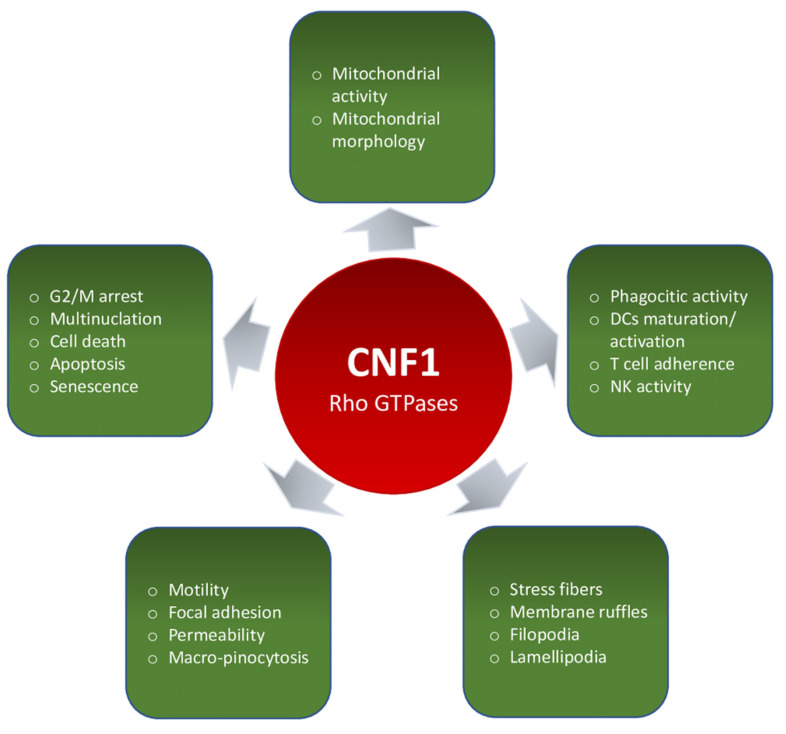
Graphical summary of the overall CNF1-induced effects reported in the literature.

CNF1 is a bacterial protein toxin mainly produced by *E. coli,* associated with extra-intestinal disease, but occasionally detected in intestinal infections [105]. For this reason, many studies have been carried out on epithelial cells that represent the actual target of the toxin, in an attempt to analyze its role in *E. coli* pathogenesis. However, due to its specific activity on Rho GTPases, CNF1 has also been used as a tool for studies aimed at deciphering the involvement of Rho GTPases in certain pathways. In the present review, we aimed at giving a comprehensive examination of the CNF1 effects described in different human and animal cell lines, in an attempt to provide an easy-to-use guide of the results obtained so far. A schematic summary of the overall CNF1-induced effects reported in the literature is shown in Figure 3.

From a general analysis of the published studies, a different activity of CNF1 does not emerge between primary, transformed and cancer cells. All in all, it is evident that the heterogeneity of the reported results reflects the great variability between the different reviewed articles in terms of experimental models, cell line tissue and type origin, experimental conditions and authors’ purpose.

Almost all the in vitro studies report the direct CNF1 enzymatic activation of Rho proteins and/or its effects on cytoskeletal organization, irrespective of the cell type and transformation status of a cell. It is also interesting to note that the depletion of Rho GTPases, ensuing CNF1 exposure, does not seem to be related to the transformation status or cell type, but rather to a specific alteration of the ubiquitin pathway in certain cells (see introduction). Rho GTPases are important transducers in signaling pathways crucial for the maintenance of normal tissue. It is well known that the same signaling pathway regulated by a specific Rho can elicit distinct responses in different cell types, depending on the biological context, such as the extracellular stimuli and signaling pathways involved in that particular cell type [93]. This is also evident from reviewing the effects of CNF1, since various TFs are activated and different proteins are regulated in many of the experimental models analyzed.

Finally, we would like to point out that none of the published papers seem to take into account the possible further consequence elicited by CNF1 interaction with its receptors, the 37 kDa LRP (37LRP) and the Lutheran adhesion glycoprotein/basal cell adhesion molecule (Lu/BCAM) [2,3]. Actually, these two distinct laminin receptors are known to be involved in a number of cellular functions, resembling those regulated by CNF1. In particular, 37LRP, acting as a mediator of cell adhesion, cell proliferation and differentiation, hampers apoptosis, plays a major role as a cell surface receptor in prion disorders and could possibly be involved in the cell biology of neurodegenerative diseases, such as Alzheimer’s disease (AD) [106,107]. In this context, it is interesting to underline that CNF1 is able to rescue cognitive deficits in a murine model of AD by increasing brain energy levels and counteracting neuroinflammatory markers [108]. Furthermore, the overexpression of 37LRP is evident in several cancer types and has been demonstrated to enhance the invasiveness of cancer cells [109].

Another aspect that could be taken into account in future studies is the possible effect due to the alteration of the balance between the F- and G-actin cellular pools, as a consequence of the CNF1-induced actin polymerization state. It has now been established that, in addition to its function in the cytoplasm, actin is actively imported into the nucleus, where it directly regulates transcription and participates in chromatin organization, mRNA transport, translation, post-translational modifications, chromosome positioning, DNA rearrangements and repair. All these functions are tightly linked to the balance between nuclear actin monomers and polymers in the nucleus and indirectly, to actin polymerization/depolymerization in the cytoplasm, which affects nuclear import/export [110].

Not least, the carcinogenic capacity of CNF1, in line with other toxins, is an emerging feature. Several modifications induced by CNF1 are, in fact, reminiscent of a procarcinogenic potential [111,112]. In recent years, studies have been carried out to corroborate this hypothesis [21,113], but the subject of study is still in its infancy.

In conclusion, although several aspects have already been addressed in studies dealing with CNF1, there are still completely new fields of investigation concerning the cellular activity of the toxin that deserve careful investigation.

## Figures and Tables

**Table 1:** Cancer cell lines.

Cell Line	Tissue Origin and Morphology	References	CNF1 Described Effects	CNF1 Preparation
T24	hu bladder, carcinoma, epithelial	[11,25,26] * [27,28] §	Rho, Rac, Cdc42, and RhoC increase Rho, Rac, and Cdc42 depletion formation of stress fibers, membrane ruffles and filopodia cell spreading and flattening increase in cell size multinucleation nuclei enlargement in mononucleated cells block of cell cycle in G2/M transition phase enhanced migration and invasion cyclin B1 reduction and cytosolic localization RhoC-dependent increase in VEGF mRNA transcription and protein secretion (under hypoxic conditions) RhoC-dependent HIF-1α protein upregulation and stabilization through the HSF1-HSP90α axis mRNA transcription and protein secretion of TNF-α, IFN-γ, IL-6, and IL-8 ROS production MMP-2 activity increase	recombinant, * purified by chromatography § His-tagged protein
UMUC3	hu bladder, carcinoma, epithelial	[28]	MMP-9 activity increase	recombinant, His-tagged protein
5637	hu bladder, carcinoma, epithelial	[26] * [27,28] §	filipodia and lamellipodia formation multinucleation increase in cell size enhanced migration and invasion block of cell cycle in G2/M transition phase cell detachment and cell death apoptosis induction MMP-2 activity increase RhoC-dependent increase in VEGF protein secretion (under hypoxic conditions) RhoC-dependent HIF-1α protein upregulation and stabilization through the HSF1-HSP90α axis	recombinant, * purified by chromatography § His-tagged protein
J82	hu bladder, carcinoma, epithelial	[27]	multinucleation increase in cell size IL-8 protein production	recombinant, His-tagged protein
SH-SY5Y	hu brain, neuroblastoma, epithelial	[29]	counteraction of the 6-OHDA-induced: − cell toxicity − phospho-Drp1 decrease − oxidative stress − mitochondrial fragmentation enrichment of the mitochondrial network autophagy induction: − increase in LC3-II expression − colocalization of LC3-II and LAMP1	recombinant, purified by chromatography
SK-N-SH	hu brain, neuroblastoma, epithelial	[30]	Rac1/AKT/NF-κB-dependent MORs protein upregulation redistribution of MORs protein at the cell surface	recombinant, purified by chromatography
U87	hu brain, glioblastoma like, epithelial	[31]	increase in SA β-gal activity p21 upregulation (mRNA)	recombinant, purified by chromatography
GBM (Glioblastoma)	hu brain, glioblastoma multiforme	[31,32]	multinucleation SA β-gal activity increase p21 upregulation (mRNA)	recombinant, purified by chromatography
MCF7	hu breast, ductal carcinoma, epithelial	[11]	Rho, Rac, and Cdc42 efficient depletion	recombinant, purified by chromatography
HeLa	hu cervix, adenocarcinoma, epithelial	[7,9,33,34,35,36,37,38]	Rho, Rac1 and Cdc42 rapid and transient activation RhoB expression proteasome-dependent Rac1 decrease formation of stress fibers, membrane ruffles, and filopodia cell spreading and flattening increase in cell size multinucleation nucleus swelling and fragmentation block of cell cycle in G2/M transition phase increase in cell-matrix binding focal adhesions formation delay in migration assays delay in the recovery of the electrical resistance after wounding activation of MAL transcription coactivator increase in AP-1 heterodimeric transcription factor activity in starved cells transient increase in JNK activity	recombinant, GST fusion protein
Caco-2	hu colon, adenocarcinoma, epithelial	[33,39,40]	Rho, Rac, and Cdc42 activation cortical actin cable elongation stress fiber and actin filament formation in focal contacts cell swelling multinucleation RhoA-dependent modulation in transepithelial resistance and in permeability of the cell monolayer	recombinant, GST fusion protein
SW480	hu colon, adenocarcinoma, epithelial	[22]	enhanced migration and invasion	recombinant, purified by chromatography
SW620	hu colon, adenocarcinoma, epithelial	[11]	Rho, Rac, and Cdc42 efficient depletion	recombinant, purified by chromatography
HT-29	hu colon, adenocarcinoma, epithelial	[22] * [37] ‡	RhoB increased expression multinucleation EMT induction: − wound healing acceleration − enhanced migration invasion − upregulation of the EMT-driving transcription factors ZEB1 and Snail1 and of vimentin − β-catenin and E-cadherin delocalization from membrane junctions to the cell body mTOR pathway activation: − lysotracker and mTOR colocalization − RagC, rpS6, and p-rpS6 increase	recombinant: * purified by chromatography ‡ GST fusion protein
T84	hu colon, adenocarcinoma, epithelial	[41,42,43,44]	Rho, Rac, and Cdc42 activation stress fiber formation cell spreading filopodia formation at the leading edge of wound margin Rho-dependent partial inhibition of wound healing effacement of brush border strong decrease of PMN transepithelial migration paracellular permeability enhancement reorganization of JAM-1 and ZO-1 away from the TJ membrane occludin internalization and colocalization with caveolin, EEA-1 and Rab11 displacement of a TJ-associated pool of phosphorylated myosin light chain effacement of microvillous F-actin and villin paxillin tyrosine phosphorylation TJ/AJ assembly impairment in calcium switch assays impairment of intercellular junction assembly permanent phosphorylation of FAK enhanced ERK, JNK, p38 phosphorylation in wounded monolayers upregulation of MMP-9 activity in wounded monolayers	recombinant, purified by chromatography
HCT-116	hu colon carcinoma, epithelial	[45]	actin filopodia formation decrease in colony formation multinucleation, endoreplication, polyploidization micronuclei associated to multinucleated cells reversible senescent arrest: − increase in SA-β-gal activity − increase in the senescence markers p53, p21, p16, HMGA-2 − decrease in pRb phosphorylation − decrease in phospho-histone H3 mitotic marker after CNF1 removal, some cells re-enter cell cycle and show: − depolyploidization − increased incidence of aneuploidy and genomic instability − enhanced resistance to CNF1	recombinant, His-tagged protein
ACHN	hu kidney, adenocarcinoma, epithelial	[27]	multinucleation increase in cell size	recombinant, His-tagged protein
A-498	hu kidney, carcinoma, epithelial	[27,28]	multinucleation increase in cell size IL-8 protein production	recombinant, His-tagged protein
HEp-2 (Human Epidermoid carcinoma #2)	hu larynx, carcinoma, epithelial	[2,7,10,21,26,45,46,47,48,49,50,51,52,53,54,55,56,57,58]	Rho and Cdc42 transient activation sustained Rac activation lower capacity of Rac ubiquitylation Rho and Cdc42 depletion formation of stress fibers, membrane ruffles and filopodia cell spreading and flattening lack of cell motility fimbrin associated to ruffles increase in cell size Rho-dependent and protein synthesis-dependent phagocytic like activity oxygen consumption increase and superoxide anion generation cell protection from UVB-induced apoptosis by improving cell–cell and cell–substrate interaction Rho- and Rac-dependent apoptosis counteraction migration and invasion enhancement multinucleation, multipolar mitosis, nuclear budding block of cell cycle in G2/M phase upregulation of cyclin B1 and p53 proteins elongated and interconnected mitochondria protection from UVB-induced mitochondrial membrane depolarization Bcl-2 and Bcl-XL proteins increase increase in mitochondrial mass NF-κB activation through: − relocalization to ruffles of Skp-1 Cullin-1-F-box containing complex and of p50/p65/IkBα − PI3k/AKT/IKK pathway activation production of CNF1 charged extracellular vesicles able to induce: − cytoskeletal changes − Rac1 and NF-κB activation increase in PdtIns-4-P 5-kinase activity relocalization of myosin 2 into stress fibers	recombinant, purified by chromatography
IGR-Heu	hu non-small-cell, lung carcinoma, epithelial	[59]	RhoA, Rac1, and Cdc42 activation Rac1 degradation	recombinant, GST fusion protein
IGR-Heu R8	hu non-small-cell, lung carcinoma, epithelial	[59]	RhoA, Rac1, and Cdc42 activation actin cytoskeleton enrichment cell spreading focal adhesions formation adhesion to type I and IV collagens increase increase in susceptibility to autologous CTL-mediated cytotoxicity FAK phosphorylation on Tyr-925 and Tyr-397	recombinant, GST fusion protein
THP-1	hu peripheral blood, acute monoblastic/monocytic leukemia, monocyte	[60] * [61] §	RhoA activation Polarized shape and F-actin content increase reduces phagocytosis of nonopsonized beads and of *E. coli* affected CR3 mediated functions modulation of CR3 activation and its colocalization with actin cytoskeleton CD36 mRNA and protein downregulation (partially through Cdc42-LXRβ signaling axis and C/EBPα)	recombinant, *purified by chromatography § His-tagged protein
JURKAT	hu peripheral blood, acute T cell leukemia, lymphoblast	[44]	Rho activation assembly of pseudopodia- and filopodia-like projections multinucleation increase in cell size increase in adherence to T84 cells monolayers	recombinant, purified by chromatography
PC3	hu prostate, adenocarcinoma, epithelial	[62] * [63] ‡	RhoA, Rac1, and Cdc42 activation enhanced migration and invasion (through Cdc42 PAK1-MMP-9 axis) PAK1 activation MMP-9 activation	recombinant, *purified by chromatography ‡ GST fusion protein
LNCaP (Lymph Node Carcinoma of the Prostate)	hu prostate, carcinoma, epithelial	[62] * [63] ‡	RhoA transient activation enhanced migration and invasion	recombinant, *purified by chromatography ‡ GST fusion protein
22Rv1	hu prostate, carcinoma, epithelial	[62]	Cdc42 activation PAK1 activation enhanced migration and invasion	recombinant, purified by chromatography
VCaP (Vertebral Cancer of the Prostate)	hu prostate, carcinoma, epithelial	[62]	enhanced migration and invasion	recombinant, purified by chromatography
Me-665	hu skin, melanoma, epithelial	[52]	formation of stress fibers, ruffles and filopodia	recombinant, purified by chromatography
RAW264.7	mouse abelson murine leukemia virus-induced tumor, monocyte/macrophage	[61]	RhoA, Rac1, and Cdc42 activation reduced phagocytosis of nonopsonized beads and of *E. coli* CD36 mRNA and protein downregulation (partially through Cdc42-LXRβ signaling axis and C/EBPα) reduction of HIF-1α mRNA levels	recombinant, His-tagged protein
Y-1	mouse adrenal cortical carcinoma, epithelial	[27]	multinucleation increase in cell size	recombinant, His-tagged protein
GL261 (Glioma 261)	mouse brain, glioblastoma, fibroblastoid	[31,32]	cell spreading and flattening multinucleation increased size of nucleoli block of cell proliferation reduction in cell migration cell death increase in SA-β-gal activity in microarray 1711 downregulated and 1318 upregulated transcripts: − downregulation of EGFR, PDGFR, and FoxG1 genes − upregulation of p16, p21, and UPP1 genes 129 upregulated proteins: − upregulation of p21 and UPP1 enrichment of functional annotated transcripts in cell cycle/senescence, DNA replication, and MAPK signaling networks pERK decrease pAKT increase	recombinant, purified by chromatography
BL6-10	mouse skin, melanoma, epithelial	[64,65]	counteraction of the inhibitory effect of simulated microgravity (SMG) on tumor growth and metastasis through: − RhoA enhanced activity − RhoA, Rac, Cdc42 and pFAK protein upregulation − restoring of cytoskeleton, focal adhesions − restoring of proliferation rates − increase in metastasis-related molecules α6β4 integrin, MMP-9 and Met72 − pAKT, pS6K and pEIF4E upregulation − pAMPK and pULK1 downregulation − mitochondrial biogenesis reduction increase in NADH and glycolytic metabolism restore focal adhesions and nuclear envelope protein complexes activation of FAK/RhoA, mTORC1/NF-κB, and ERK1/2 pathways, leading to reduced apoptosis in cells under SMG	recombinant, GST-fusion protein
B16-F10	mouse skin, melanoma, mixture of spindle-shaped and epithelial-like	[66]	stress fiber formation inhibition of cAMP-promoted dendrite outgrowth decrease of the forskolin-induced stimulation of luciferase promoter activity decrease of both basal- and forskolin-induced increase in tyrosinase protein expression	not specified
804G	rat bladder, carcinoma, epithelial	[8,11]	RhoA, Rac1 and Cdc42 transient activation reversible and proteasome-dependent depletion of RhoA, Rac, and Cdc42 Rac ubiquitylation increase partial relocalization of Rac from the cytosol to the plasma membrane and perinuclear vesicles cell spreading followed by cell retraction filamentous actin increase cell motility induction Rac-dependent uropathogenic bacteria invasion induction	recombinant, purified by chromatography

* purified by chromatography; § His-tagged protein; ‡ GST fusion protein

**Table 2:** Immortalized/transformed cell lines

Cell Line	Tissue Origin and Morphology	References	CNF1 Described Effects	CNF1 Preparation
HMEC-1 (Human Microvascular Endothelial Cell line-1)	hu dermal endothelium, immortalized (SV40 T-antigen), endothelial-like	[67]	stress fiber formation ZO-1, VE-cadherin and β-catenin increase stronger interendothelial adhesion transendothelial permeability reduction decrease of monocyte transmigration through HMEC-1 monolayer	recombinant, GST fusion protein
HEK 293 (Human Embryonic Kidney 293)	hu embryonic kidney, transformed, tumorigenic, epithelial	[11] * [9] ‡	Cdc42 sustained activation Rho depletion proteasome-dependent Rac1 depletion actin filopodia formation transient increase in JNK activity	recombinant, * purified by chromatography ‡ GST fusion protein
HEK 293T (Human Embryonic Kidney 293T)	hu embryonic kidney, transformed, HEK 293 derivative expressing SV40 T-antigen, epithelial	[68]	NF-κB activation and IL-8 expression dependent on Rac2 activation and Rip1 and Rip2 adaptor proteins	recombinant, His-tagged protein
HBMEC-60 (Human Bone Marrow Endothelial Cell line-60)	hu bone marrow, immortalized (HPV16 E6/E7), endothelial	[69]	decrease of pRBC cytoadherence of *P. falciparum* (prebinding assay) reversing of pRBC cytoadherence of *P. falciparum* (postbinding assay)	recombinant, purified by chromatography
HBEC-5i (Human Brain Endothelial Cell line-5i)	hu brain cerebral cortex, immortalized (SV40 T-antigen), endothelial	[22,69]	RhoA, Rac1, and Cdc42 activation stress fiber and filopodia formation cell spreading and flattening decrease of cell motility decrease of pRBC cytoadherence of *P. falciparum* (pre-binding assay) reversing of pRBC cytoadherence of *P. falciparum* (post-binding assay) ICAM-1 decreased expression counteraction of the pRBCs-induced monolayer permeability	recombinant, purified by chromatography
SV-HUC-1	hu ureter, immortalized (SV40 T-antigen), epithelial	[27]	multinucleation	recombinant, His-tagged protein
MesEnd (Mesenteric Endothelial)	mouse mesenteric microvascular, immortalized (SV40 T-antigen),	[70]	Rac1, Cdc42, and RhoA activation stress fiber formation	recombinant, GST fusion protein
MyEnd (Myocardial Endothelial)	mouse microvascular myocardial, immortalized (SV40 T-antigen)	[70,71,72]	Rac1 and Cdc42 activation increase in filaments in the junction-associated actin belt decrease in monolayer permeability Rac1-dependent redistribution of cortactin and VASP to cell border VASP localization to cell junction VASP colocalization with VE-cadherin and ZO-1 cortactin redistribution to cell borders	recombinant, GST fusion protein
HaCaT	hu skin, spontaneously immortalized, keratinocyte	[73]	strengthening of the peripheral junction-associated actin belt abrogation of PV-IgG-induced loss of cell adhesion block of PV-IgG-mediated Dsg3 fragmentation block of PV-IgG-mediated actin remodeling	not specified
NIH 3T3	mouse embryonic, spontaneously immortalized, fibroblasts	[6,33,37,74]	RhoA, RhoB, Rac1, and Cdc42 activation RhoA, Rac1, and Cdc42 depletion Rac-dependent increase in RhoB protein level lamellipodia formation multinucleation DNA synthesis stimulation cytotoxicity c-Myc expression Rac1-c-Myc-dependent increase in RhoB expression (protein and mRNA) Rac1-c-Myc-dependent activation of RhoB promoter	recombinant, GST fusion protein
3T3-Swiss albino	mouse embryo, spontaneously immortalized, fibroblasts	[53]	cytotoxicity binucleation	recombinant, His-tagged protein
3T3-L1	mouse embryo, substrain of 3T3-Swiss albino, preadipocytes fibroblasts	[75]	stress fiber formation cell spreading multinucleation block in cell proliferation block of differentiation (adipogenesis): − block of the induction of PPARγ and C/EBPα expression − maintaining of elevated levels of Pref1/Dlk1 and β-catenin downregulation of Notch1 protein expression	recombinant, His-tagged protein
C2C12	mouse muscle, spontaneously immortalized, myoblast	[76]	Rho, Rac, and Cdc42 activation stress fiber formation myotube formation impairment Rho-dependent downregulation of MHC, MyoD and myogenin expression in differentiation medium (protein and mRNA)	recombinant, purified by chromatography
Vero	monkey kidney, spontaneously immortalized, epithelial	[5,11,56]	Rho sustained activation Rac1 and Cdc42 depletion stress fiber formation limited membrane ruffles cell spreading	recombinant, purified by chromatography
MDCK (Madin-Darby Canine Kidney)	dog kidney, spontaneously immortalized, epithelial	[77]	increased *P. aeruginosa* internalization ZO-1 dislocation from tight junctions to cytoplasm E-cadherin aberrant redistribution in both apical and basolateral membranes	recombinant, GST fusion protein
PAE (p23 clone) (Porcine Aorta-derived Endothelial)	pig aorta	[78]	transient activation of RhoA sustained activation of Rac1 ana Cdc42 Cdc42-dependent assembly of F-actin in podosomes	recombinant, GST fusion protein

* purified by chromatography; ‡ GST fusion protein

**Table 3:** Finite/primary cell lines

Cell line	Tissue Origin and Morphology	References	CNF1 Described Effects	CNF1 Preparation
HPECC (HPCEC)	hu colon, finite cell line, epithelial	[22]	cell motility decrease	recombinant, purified by chromatography
HDMEC (Human Dermal Microvascular Endothelial Cells)	hu dermal, finite cell line, microvascular endothelial cells	[70,79]	Rac1, Cdc42, and RhoA activation Cdc42 protein increase stress fiber formation marked peripheral actin Rac1 and cortactin translocation to cell junctions enhanced claudin 5 immunostaining intensity tight junction linearization slight decrease of monolayer permeability	recombinant, GST fusion protein
HUVEC (Human Umbilical Vein Endothelial Cell)	hu umbilical, finite cell line, endothelial	[8,10,11,28,80]	Rho, Rac, and Cdc42 transient activation Rho, Rac, and Cdc42 efficient depletion stress fiber formation F-actin accumulation at junctional borders enhanced migration and invasion protection from barrier-disruptive agents (thrombin) p38 MAPK and c-Jun phosphorylation IκB-α depletion Rac- and Cdc42-dependent induction of inflammatory mediators-encoding genes (microarray) E-selectin, MCP-1, MIP-3α, IL-8, IL-6, TRAF1 proteins increase	recombinant, purified by chromatography, His-tagged protein
IEC-6 (Intestinal Epithelioid Cell line #6)	rat small intestine, finite cell line, epithelial	[22,81]	transient Rho, Rac, and Cdc42 activation ATP production increase increase in the activity of complex V (ATP synthase) Rho- and Rac-dependent enrichment of mitochondrial network (mitochondria elongation) Bcl-2 protein expression decrease Drp1 phosphorylation (Ser637) cAMP content increase PKA activity increase vimentin expression decrease in the presence of supernatant from activated immune cells: − cell motility increase − transient Snail1 protein upregulation	recombinant, purified by chromatography
T-lymphocytes	hu blood, primary, lymphocyte	[44]	Rho activation F-actin content increase formation of pseudopodia and filopodia-like projections clustering of CD29, CD11a and CD49d integrins into filopodia enhanced SDF1α-induced migration across acellular filters enhanced adherence to epithelial cells disruption of the epithelial cell monolayer transient p42-44MAPK and JNK activation TNF-α and TGF-β mRNA increase TNF-α and TGF-β secretion increase	recombinant, purified by chromatography
NK	hu blood, primary, large granular lymphocyte	[82]	transient Rac activation increased F-actin polarization in contact region between NK and NK-target cell increased cytotoxicity increased binding to the target cell recruitment of a higher number of effector cells on the same target cell increased CD69, CD18, ICAM-1, IL-2R, and HLA-DR proteins	recombinant, purified by chromatography
monocytes	hu blood, primary, monocyte	[60]	Rho activation lamellipodia and knob-like protuberance formation cell spreading disorganization of actin microfilaments (concentration of F-actin in foci) actin cable formation decreased ingestion of unopsonized zymosan (CR3 mediated) modulation of CR3 activation of and its colocalization with actin cytoskeleton clustering of CD11b, CD32 and CD18 in peripheral patches decreased colocalization of CD11b, CD18, and CR3 with F-actin	recombinant, purified by chromatography
macrophages	hu blood, primary, macrophage	[11]	Rho, Rac, and Cdc42 depletion	recombinant, purified by chromatography
DC (dendritic cell) monocytes	hu blood, primary, monocyte	[83]	phenotypic and functional maturation of moDCs: − increased CD83 and CD86 double-positive cell number − increased surface expression of HLA-DR MHC class II molecules − increased secretion of IL-6 and TNF-α − increased capacity to induce proliferation of allogenic naïve CD4+ T-lymphocytes	recombinant, purified by chromatography
HBMEC (Human Brain Microvascular Endothelial Cells)	hu brain, primary, microvascular endothelium	[3]	RhoA activation increased *E. coli* E44 invasion	recombinant, GST fusion protein
keratinocytes	hu neonatal foreskin, primary, keratinocyte	[11]	RhoA, Rac1, and Cdc42 depletion	recombinant, purified by chromatography
MERRF (Myoclonic Epilepsy with Ragged-Red Fibers) fibroblasts	hu skin, primary, from myoclonic epilepsy with ragged-red fibers, fibroblast	[84]	increase in stress fiber number and thickness, rescuing wild-type phenotype rescue of the mitochondrial morphology ATP content increase Tom20 expression increase	recombinant, purified by chromatography
fibroblasts	hu skin/neonatal foreskin, primary, fibroblast	[11,84]	RhoA, Rac1, and Cdc42 depletion stress fiber increase ATP content increase Tom20 expression increase	recombinant, purified by chromatography
BMDM (Bone-Marrow-Derived Macrophages)	mouse bone marrow derived macrophages	[85] * [61] §	reduced phagocytosis of nonopsonized beads and of *E. coli* CD36 mRNA and protein downregulation (partially through Cdc42-LXRβ signaling axis and C/EBPα) NLRP3 inflammasome activation (through Rac2 and PAK1) caspase-1 activation IL-1β protein maturation and secretion	recombinant, * purified by chromatography § His-tagged protein
MEFs (Mouse Embryonic Fibroblasts)	mouse embryo, primary, fibroblast	[11]	transient RhoA activation RhoA, Rac1, and Cdc42 depletion	recombinant, purified by chromatography
mouse peritoneal macrophages	mouse peritoneal lavage, primary, macrophage	[61]	reduced phagocytosis of nonopsonized *E. coli* CD36 mRNA and protein downregulation (partially through Cdc42-LXRβ signaling axis and C/EBPα)	recombinant, His-tagged protein
rat mesangial primary cells	rat kidney, primary	[86]	increase of Cox2 mRNA levels	recombinant, His-tagged protein
rat embryonic primary astrocytes	rat embryo cortex, primary, astrocytes	[87]	reduction of GFAP protein levels reduction of IL-1β levels reduction of glutamate-dependent intracellular Ca2+ rise transformation of astrocytes in an efficient substrate for neuritogenesis and synaptogenesis (in vitro)	recombinant, purified by chromatography
rat embryonic primary neurons	rat embryonic hippocampus	[87]	partially reversible block of neuronal differentiation (less evident in in the presence of astrocytes): − development of filopodia-like protrusions along neurites and around cell bodies − thick and tortuous dendrite formation − lack of synapse formation and reduced synaptic density − poor dendritic branching − colocalization of pre- (synaptophysin) and post-synaptic markers (PSD95) on differentiated neurons: − synapse remodeling − decreased synaptophysin positive dots	recombinant, purified by chromatography
rat embryonic primary neurons	rat newborn hippocampus	[88]	dendrite and axon retraction	recombinant, GST-fusion
rat embryonic primary neurons	rat embryonic substantia nigra	[89]	increase in neuronal process length and complexity activation of structural plasticity	recombinant, His-SUMO tag protein
OPC (Oligodendrocyte Precursor Cells)	rat/mouse newborn cortex, primary	[90]	RhoA and Rac1 activation increase of myelin sheet formation	recombinant, GST fusion protein
PAEC (Porcine Aorta Endothelial Cells)	pig pulmonary artery, primary, endothelial	[70]	RhoA. Rac1, and Cdc42 activation stress fiber formation increased peripheral F-actin staining VE-cadherin fragmentation intercellular gap formation Rho-dependent increase in cell monolayer permeability	recombinant, GST fusion protein
dog thyroid epithelial cells	dog thyroid, primary, epithelial-like	[91]	Rac1 and Cdc42 activation Rac1 depletion rescue from forskolin-induced stress fiber disruption counteraction of forskolin-dependent induction of thyroid differentiation genes (Tg, NIS, and ThOXs)	recombinant, GST fusion protein

* purified by chromatography; § His-tagged protein

## Supplementary Materials

The following are available online at https://www.mdpi.com/article/10.3390/ijms222212610/s1.

## Abbreviations

AKT	RAC- serine/threonine-protein kinase
AMPK	5′-AMP-activated protein kinase catalytic subunit alpha-2
AP-1	activator protein 1
Bcl-2	apoptosis regulator Bcl-2 (B-cell lymphoma 2)
Bcl-XL	apoptosis regulator Bcl-X (B-cell lymphoma-extra-large)
C/EBPα	CCAAT/enhancer-binding protein alpha
cAMP	cyclic adenosine monophosphate
CD11a	integrin alpha-L
CD11b	integrin alpha-M
CD18	integrin beta-2
CD29	integrin beta-1
CD32	low affinity immunoglobulin gamma Fc region receptor II-b
CD36	platelet glycoprotein 4
CD49d	integrin alpha-4
CD83	cluster of differentiation 83 antigen
CD86	T-lymphocyte activation antigen CD86
CD96	T-cell surface protein tactile
c-Myc	proto-oncogene protein Myc
c-Jun	AP-1 transcription factor subunit
CR3	complement receptor 3 C
DLK1	protein delta homolog 1
Drp1	dynamin-1-like protein
Dsg3	desmoglein-3
EEA-1	early endosome antigen 1
EGFR	epidermal growth factor receptor
EIF4E	eukaryotic translation initiation factor 4E
EMT	epithelial–mesenchymal transition
ERK1/2, (p42-44, MAPK)	mitogen-activated protein kinases 3 and 1
FAK	focal adhesion kinase
FoxG1	forkhead box protein G1
GFAP	glial fibrillary acidic protein
HIF-1α	hypoxia-inducible factor 1-alpha
HLA-DR	HLA class II histocompatibility antigen gamma chain
HMGA-2	high mobility group protein HMGI-C
HSF1	heat shock factor protein 1
HSP90α	heat shock protein HSP 90-alpha
ICAM-1	intercellular adhesion molecule 1
IFN-γ	interferon gamma
IL-1β	interleukin 1 beta
IL-2R	interleukin 2 receptor
IL-6	interleukin 6
IL-8	interleukin 8
IκB-α, IKK	NF-κB inhibitor alpha
JAM-1	junctional adhesion molecule A
JNK	mitogen-activated protein kinase 8
LAMP1	lysosome-associated membrane glycoprotein 1
LC3-II	microtubule-associated proteins 1A/1B light chain 3B
LXRβ	liver X receptor beta
MAL	myocardin-related transcription factor A
MCP-1	monocyte chemoattractant protein 1
Met72	melanoma cell-surface 72 Kd-glycoprotein
MHC	major histocompatibility complex
MIP-3α	macrophage inflammatory protein-3 alpha
MMP-2	matrix metalloproteinase-2
MMP-9	matrix metalloproteinase-9
moDC	monocyte-derived dendritic cells
MORs	mu-opioid receptors
mTOR	mammalian target of rapamycin
mTORC1	mTOR complex 1
MyoD	myoblast determination protein 1
NADH	nicotinamide adenine dinucleotide, reduced form
NF-κB	nuclear factor kappa-light-chain-enhancer of activated B cells
NIS	sodium/iodide cotransporter
Notch1	neurogenic locus notch homolog protein 1
p16	cyclin-dependent kinase inhibitor 2A
p21	cyclin-dependent kinase inhibitor 1
p38	mitogen-activated protein kinase 11
p53	cellular tumor antigen p53
PAK1	serine/threonine-protein kinase PAK1
pAKT	RAC- serine/threonine-protein kinase, phosphorylate form
PDGFR	platelet-derived growth factor receptor
PdtIns-4-P 5-kinase	phosphatidylinositol 4-phosphate 5-kinase
PKA	cAMP-dependent protein kinase
PMN	polymorphonuclear neutrophils
PPARγ	peroxisome proliferator-activated receptor gamma
pRb	retinoblastoma-associated protein
pRBC	*Plasmodium falciparum*-parasitized erythrocytes
Pref1	preadipocyte factor
pS6K	ribosomal protein S6 kinase, phosphorylate form
pULK1	unc-51 like autophagy activating kinase 1, phosphorylate form
PV-IgG	pemphigus vulgaris autoantibodies
Rab11	Ras-related protein Rab-11
RagC	Ras-related GTP-binding protein C
rpS6	40S ribosomal protein S6
Rip1, Rip2	receptor-interacting serine/threonine-protein kinase 1 and 2
ROS	reactive oxygen species
SA-β-gal	senescence-associated beta-galactosidase
SDF-1α	stromal cell-derived factor 1
SMG	simulated microgravity
Snail1	snail family transcriptional repressor 1
TF	transcription factor
Tg	thyroglobulin
ThOX	thyroid oxidase
TGF-β	transforming growth factor beta
TJ/AJ	tight junction/adherent junction
TNF-α	tumor necrosis factor alpha
Tom20	mitochondrial import receptor subunit
TRAF1	TNF receptor-associated factor 1
UPP1	uridine-phosphorylase 1
VEGF	vascular endothelial growth factor
VASP	vasodilator-stimulated phosphoprotein
ZEB1	zinc finger E-box-binding homeobox 1
ZO-1	Zonula occudens-1 (tight junction protein 1)

## References

[1] Boquet P. (2001). The cytotoxic necrotizing factor 1 (CNF1) from Escherichia coli. Toxicon.

[2] Piteau M., Papatheodorou P., Schwan C., Schlosser A., Aktories K., Schmidt G. (2014). Lu/BCAM Adhesion Glycoprotein Is a Receptor for Escherichia coli Cytotoxic Necrotizing Factor 1 (CNF1). PLoS Pathog..

[3] Chung J.W., Hong S.J., Kim K.J., Goti D., Stins M.F., Shin S., Dawson V.L., Dawson T.M., Kim K.S. (2003). 37-kDa laminin receptor precursor modulates cytotoxic necrotizing factor 1-mediated RhoA activation and bacterial uptake. J. Biol. Chem..

[4] Contamin S., Galmiche A., Doye A., Flatau G., Benmerah A., Boquet P. (2000). The p21 Rho-activating toxin cytotoxic necrotizing factor 1 is endocytosed by a clathrin-independent mechanism and enters the cytosol by an acidic-dependent membrane translocation step. Mol. Biol. Cell.

[5] Flatau G., Lemichez E., Gauthier M., Chardin P., Paris S., Fiorentini C., Boquet P. (1997). Toxin-induced activation of the G protein p21 Rho by deamidation of glutamine. Nature.

[6] Schmidt G., Sehr P., Wilm M., Selzer J., Mann M., Aktories K. (1997). Gln 63 of Rho is deamidated by Escherichia coli cytotoxic necrotizing factor-1. Nature.

[7] Lerm M., Selzer J., Hoffmeyer A., Rapp U.R., Aktories K., Schmidt G. (1999). Deamidation of Cdc42 and Rac by Escherichia coli cytotoxic necrotizing factor 1: Activation of c-Jun N-terminal kinase in HeLa cells. Infect. Immun..

[8] Doye A., Mettouchi A., Bossis G., Clément R., Buisson-Touati C., Flatau G., Gagnoux L., Piechaczyk M., Boquet P., Lemichez E. (2002). CNF1 exploits the ubiquitin-proteasome machinery to restrict Rho GTPase activation for bacterial host cell invasion. Cell.

[9] Lerm M., Pop M., Fritz G., Aktories K., Schmidt G. (2002). Proteasomal degradation of cytotoxic necrotizing factor 1-activated rac. Infect. Immun..

[10] Munro P., Flatau G., Doye A., Boyer L., Oregioni O., Mege J.-L., Landraud L., Lemichez E. (2004). Activation and Proteasomal Degradation of Rho GTPases by Cytotoxic Necrotizing Factor-1 Elicit a Controlled Inflammatory Response. J. Biol. Chem..

[11] Boyer L., Turchi L., Desnues B., Doye A., Ponzio G., Mege J.L., Yamashita M., Zhang Y.E., Bertoglio J., Flatau G. (2006). CNF1-induced ubiquitylation and proteasome destruction of activated RhoA is impaired in Smurf1−/− cells. Mol. Biol. Cell.

[12] Hodge R.G., Ridley A.J. (2016). Regulating Rho GTPases and their regulators. Nat. Rev. Mol. Cell Biol..

[13] Ridley A. (2006). Rho GTPases and actin dynamics in membrane protrusions and vesicle trafficking. Trends Cell Biol..

[14] Duquette P., Lamarche-Vane N. (2014). Rho GTPases in embryonic development. Small GTPases.

[15] Huang G., Sun Z., Li H., Feng D. (2017). Rho GTPase-activating proteins: Regulators of Rho GTPase activity in neuronal development and CNS diseases. Mol. Cell. Neurosci..

[16] Niftullayev S., Lamarche-Vane N. (2019). Regulators of rho GTPases in the nervous system: Molecular implication in axon guidance and neurological disorders. Int. J. Mol. Sci..

[17] Chai L., Cao C., Bi S., Dai X., Gan L., Guo R., Li R. (2010). Small Rho GTPase Rac1 determines human epidermal stem cell fate in vitro. Int. J. Mol. Med..

[18] Yang L., Wang L., Geiger H., Cancelas J., Mo J., Zheng Y. (2007). Rho GTPase Cdc42 coordinates hematopoietic stem cell quiescence and niche interaction in the bone marrow. Proc. Natl. Acad. Sci. USA.

[19] Bros M., Haas K., Moll L., Grabbe S. (2019). RhoA as a Key Regulator of Innate and Adaptive Immunity. Cells.

[20] Etienne-Manneville S., Hall A. (2002). Rho GTPases in cell biology. Nature.

[21] Popoff M.R. (2014). Bacterial factors exploit eukaryotic Rho GTPase signaling cascades to promote invasion and proliferation within their host. Small GTPases.

[22] Fabbri A., Travaglione S., Rosadi F., Ballan G., Maroccia Z., Giambenedetti M., Guidotti M., Ødum N., Krejsgaard T., Fiorentini C. (2019). The Escherichia coli protein toxin cytotoxic necrotizing factor 1 induces epithelial mesenchymal transition. Cell Microbiol..

[23] Buc E., Dubois D., Sauvanet P., Raisch J., Delmas J., Darfeuille-Michaud A., Pezet D., Bonnet R. (2013). High Prevalence of Mucosa-Associated, E. coli Producing Cyclomodulin and Genotoxin in Colon Cancer. PLoS ONE.

[24] Moher D., Liberati A., Tetzlaff J., Altman D.G. (2009). Preferred reporting items for systematic reviews and meta-analyses: The PRISMA statement David Moher and colleagues introduce PRISMA, an update of the QUOROM guidelines for reporting systematic reviews and meta-analyses. BMJ.

[25] Falzano L., Quaranta M.G., Travaglione S., Filippini P., Fabbri A., Viora M., Donelli G., Fiorentini C. (2003). Cytotoxic necrotizing factor 1 enhances reactive oxygen species-dependent transcription and secretion of proinflammatory cytokines in human uroepithelial cells. Infect. Immun..

[26] Falzano L., Filippini P., Travaglione S., Miraglia A.G., Fabbri A., Fiorentini C. (2006). Escherichia coli cytotoxic necrotizing factor 1 blocks cell cycle G2/M transition in uroepithelial cells. Infect. Immun..

[27] Mills M., Meysick K.C., O’Brien A.D. (2000). Cytotoxic necrotizing factor type 1 of uropathogenic Escherichia coli kills cultured human uroepithelial 5637 cells by an apoptotic mechanism. Infect. Immun..

[28] Guo Y., Wang J., Zhou K., Lv J., Wang L., Gao S., Keller E.T., Zhang Z.S., Wang Q., Yao Z. (2020). Cytotoxic necrotizing factor 1 promotes bladder cancer angiogenesis through activating RhoC. FASEB J..

[29] Travaglione S., Loizzo S., Vona R., Ballan G., Rivabene R., Giordani D., Guidotti M., Dupuis M.L., Maroccia Z., Baiula M. (2020). The Bacterial Toxin CNF1 Protects Human Neuroblastoma SH-SY5Y Cells against 6-Hydroxydopamine-Induced Cell Damage: The Hypothesis of CNF1-Promoted Autophagy as an Antioxidant Strategy. Int. J. Mol. Sci..

[30] Pavone F., Luvisetto S., Marinelli S., Straface E., Fabbri A., Falzano L., Fiorentini C., Malorni W. (2009). The Rac GTPase-activating bacterial protein toxin CNF1 induces analgesia up-regulating mu-opioid receptors. Pain.

[31] Vannini E., Olimpico F., Middei S., Ammassari-Teule M., de Graaf E.L., McDonnell L., Schmidt G., Fabbri A., Fiorentini C., Baroncelli L. (2016). Electrophysiology of glioma: A Rho GTPase-activating protein reduces tumor growth and spares neuron structure and function. Neuro. Oncol..

[32] Vannini E., Panighini A., Cerri C., Fabbri A., Lisi S., Pracucci E., Benedetto N., Vannozzi R., Fiorentini C., Caleo M. (2014). The bacterial protein toxin, cytotoxic necrotizing factor 1 (CNF1) provides long-term survival in a murine glioma model. BMC Cancer.

[33] Stoll T., Markwirth G., Reipschläger S., Schmidt G. (2009). A new member of a growing toxin family--Escherichia coli cytotoxic necrotizing factor 3 (CNF3). Toxicon.

[34] Huelsenbeck S., May M., Schmidt G., Genth H. (2009). Inhibition of cytokinesis by Clostridium difficile toxin B and cytotoxic necrotizing factors--reinforcing the critical role of RhoA in cytokinesis. Cell Motil. Cytoskeleton.

[35] Dong N., Liu L., Shao F. (2010). A bacterial effector targets host DH-PH domain RhoGEFs and antagonizes macrophage phagocytosis. EMBO J..

[36] May M., Kolbe T., Wang T., Schmidt G., Genth H. (2012). Increased Cell-Matrix Adhesion upon Constitutive Activation of Rho Proteins by Cytotoxic Necrotizing Factors from E. Coli and Y. Pseudotuberculosis. J. Signal Transduct..

[37] Huelsenbeck S.C., Roggenkamp D., May M., Huelsenbeck J., Brakebusch C., Rottner K., Ladwein M., Just I., Fritz G., Schmidt G. (2013). Expression and cytoprotective activity of the small GTPase RhoB induced by the Escherichia coli cytotoxic necrotizing factor 1. Int. J. Biochem. Cell Biol..

[38] Pfaumann V., Lang A.E., Schwan C., Schmidt G., Aktories K. (2015). The actin and Rho-modifying toxins PTC3 and PTC5 of Photorhabdus luminescens: Enzyme characterization and induction of MAL/SRF-dependent transcription. Cell. Microbiol..

[39] Gerhard R., Schmidt G., Hofmann F., Aktories K. (1998). Activation of Rho GTPases by Escherichia coli cytotoxic necrotizing factor 1 increases intestinal permeability in Caco-2 cells. Infect. Immun..

[40] Schlegel N., Meir M., Spindler V., Germer J., Wascher J. (2011). Differential role of Rho GTPases in intestinal epithelial barrier regulation in vitro. J. Cell. Physiol..

[41] Hofman P., Flatau G., Selva E., Gauthier M., Le Negrate G., Fiorentini C., Rossi B., Boquet P. (1998). Escherichia coli cytotoxic necrotizing factor 1 effaces microvilli and decreases transmigration of polymorphonuclear leukocytes in intestinal T84 epithelial cell monolayers. Infect. Immun..

[42] Hopkins A., Walsh S., Verkade P., Boquet P., Nusrat A. (2003). Constitutive activation of Rho proteins by CNF-1 influences tight junction structure and epithelial barrier function. J. Cell Sci..

[43] Brest P., Turchi L., Le’Negrate G., Berto F., Moreilhon C., Mari B., Ponzio G., Hofman P. (2004). Escherichia coli cytotoxic necrotizing factor 1 inhibits intestinal epithelial wound healing in vitro after mechanical injury. Infect. Immun..

[44] Brest P., Mograbi B., Hofman V., Loubat A., Rossi B., Auberger P., Hofman P. (2003). Rho GTPase is activated by cytotoxic necrotizing factor 1 in peripheral blood T lymphocytes: Potential cytotoxicity for intestinal epithelial cells. Infect. Immun..

[45] Zhang Z., Aung K.M., Uhlin B.E., Wai S.N. (2018). Reversible senescence of human colon cancer cells after blockage of mitosis/cytokinesis caused by the CNF1 cyclomodulin from Escherichia coli. Sci. Rep..

[46] Falzano L., Fiorentini C., Donelli G., Michel E., Kocks C., Cossart P., Cabanié L., Oswald E., Boquet P. (1993). Induction of phagocytic behaviour in human epithelial cells by Escherichia coli cytotoxic necrotizing factor type 1. Mol. Microbiol..

[47] Fiorentini C., Donelli G., Matarrese P., Fabbri A., Paradisi S., Boquet P. (1995). Escherichia coli cytotoxic necrotizing factor 1: Evidence for induction of actin assembly by constitutive activation of the p21 Rho GTPase. Infect. Immun..

[48] Fiorentini C., Fabbri A., Flatau G., Donelli G., Matarrese P., Lemichez E., Falzano L., Boquet P. (1997). Escherichia coli cytotoxic necrotizing factor 1 (CNF1), a toxin that activates the Rho GTPase. J. Biol. Chem..

[49] Fiorentini C., Matarrese P., Straface E., Falzano L., Donelli G., Boquet P., Malorni W. (1998). Rho-dependent cell spreading activated by E.coli cytotoxic necrotizing factor 1 hinders apoptosis in epithelial cells. Cell Death Differ..

[50] Falzano L., Rivabene R., Santini M.T., Fabbri A., Fiorentini C. (2001). An Escherichia coli cytotoxin increases superoxide anion generation via Rac in epithelial cells. Biochem. Biophys. Res. Commun..

[51] Fiorentini C., Falzano L., Fabbri A., Stringaro A., Logozzi M., Travaglione S., Contamin S., Arancia G., Malorni W., Fais S. (2001). Activation of Rho GTPases by Cytotoxic Necrotizing Factor 1 Induces Macropinocytosis and Scavenging Activity in Epithelial Cells. Mol. Biol. Cell.

[52] Falzano L., Rivabene R., Fabbri A., Fiorentini C. (2002). Epithelial cells challenged with a Rac-activating E. coli cytotoxin acquire features of professional phagocytes. Toxicol. Vitr..

[53] Boyer L., Travaglione S., Falzano L., Gauthier N.C., Popoff M.R., Lemichez E., Fiorentini C., Fabbri A. (2004). Rac GTPase Instructs Nuclear Factor-κB Activation by Conveying the SCF Complex and IkBα to the Ruffling Membranes. Mol. Biol. Cell.

[54] Malorni W., Fiorentini C. (2006). Is the Rac GTPase-activating toxin CNF1 a smart hijacker of host cell fate?. FASEB J..

[55] Giamboi-Miraglia A., Travaglione S., Filippini P., Fabbri A., Fiorentini C., Falzano L. (2007). A multinucleating Escherichia coli cytotoxin perturbs cell cycle in cultured epithelial cells. Toxicol. Vitro.

[56] Miraglia A.G., Travaglione S., Meschini S., Falzano L., Matarrese P., Quaranta M.G., Viora M., Fiorentini C., Fabbri A. (2007). Cytotoxic necrotizing factor 1 prevents apoptosis via the Akt/IkappaB kinase pathway: Role of nuclear factor-kappaB and Bcl-2. Mol. Biol. Cell.

[57] Fabbri A., Cori S., Zanetti C., Guidotti M., Sargiacomo M., Loizzo S., Fiorentini C. (2015). Cell-to-cell propagation of the bacterial toxin CNF1 via extracellular vesicles: Potential impact on the therapeutic use of the toxin. Toxins.

[58] McNichol B.A., Rasmussen S.B., Meysick K.C., O’Brien A.D. (2006). A single amino acid substitution in the enzymatic domain of cytotoxic necrotizing factor type 1 of Escherichia coli alters the tissue culture phenotype to that of the dermonecrotic toxin of Bordetella spp. Mol. Microbiol..

[59] Abouzahr-Rifai S., Hasmim M., Boukerche H., Hamelin J., Janji B., Jalil A., Kieda C., Mami-Chouaib F., Bertoglio J., Chouaib S. (2008). Resistance of tumor cells to cytolytic T lymphocytes involves Rho-GTPases and focal adhesion kinase activation. J. Biol. Chem..

[60] Capo C., Meconi S., Sanguedolce M.V., Bardin N., Flatau G., Boquet P., Mege J.L. (1998). Effect of cytotoxic necrotizing factor-1 on actin cytoskeleton in human monocytes: Role in the regulation of integrin-dependent phagocytosis. J. Immunol..

[61] Yang H., Li Q., Wang C., Wang J., Lv J., Wang L., Zhang Z.S., Yao Z., Wang Q. (2018). Cytotoxic Necrotizing Factor 1 Downregulates CD36 Transcription in Macrophages to Induce Inflammation During Acute Urinary Tract Infections. Front. Immunol..

[62] Guo Y., Zhang Z., Wei H., Wang J., Lv J., Zhang K., Keller E.T., Yao Z., Wang Q. (2017). Cytotoxic necrotizing factor 1 promotes prostate cancer progression through activating the Cdc42-PAK1 axis. J. Pathol..

[63] Augspach A., List J., Wolf P., Bielek H., Schwan C., Elsässer-Beile U., Aktories K., Schmidt G. (2013). Activation of RhoA,B,C by Yersinia Cytotoxic Necrotizing Factor (CNFy) induces apoptosis in LNCaP prostate cancer cells. Toxins.

[64] Tan X., Xu A., Zhao T., Zhao Q., Zhang J., Fan C., Deng Y., Freywald A., Genth H., Xiang J. (2018). Simulated microgravity inhibits cell focal adhesions leading to reduced melanoma cell proliferation and metastasis via FAK/RhoA-regulated mTORC1 and AMPK pathways. Sci. Rep..

[65] Zhao T., Li R., Tan X., Zhang J., Fan C., Zhao Q., Deng Y., Xu A., Lukong K.E., Genth H. (2018). Simulated microgravity reduces focal adhesions and alters cytoskeleton and nuclear positioning leading to enhanced apoptosis via suppressing FAK/Rhoa-mediated mTORC1/NF-κB and ERK1/2 pathways. Int. J. Mol. Sci..

[66] Buscà R., Bertolotto C., Abbe P., Englaro W., Ishizaki T., Narumiya S., Boquet P., Ortonne J.P., Ballotti R. (1998). Inhibition of Rho is required for cAMP-induced melanoma cell differentiation. Mol. Biol. Cell.

[67] Stray A., Janning A., Barth H., Gerke V. (2002). Endothelial Rho signaling is required for monocyte transendothelial migration. FEBS Lett..

[68] Boyer L., Magoc L., Dejardin S., Cappillino M., Paquette N., Hinault C., Charriere G., Ip W., Fracchia S., Hennessy E. (2011). Pathogen-derived effectors trigger protective immunity via activation of the Rac2 enzyme and the IMD or Rip kinase signaling pathway. Immunity.

[69] Messina V., Loizzo S., Travaglione S., Bertuccini L., Condello M., Superti F., Guidotti M., Alano P., Silvestrini F., Fiorentini C. (2019). The bacterial protein CNF1 as a new strategy against Plasmodium falciparum cytoadherence. PLoS ONE.

[70] Baumer Y., Burger S., Curry F.E., Golenhofen N., Drenckhahn D., Waschke J. (2008). Differential role of Rho GTPases in endothelial barrier regulation dependent on endothelial cell origin. Histochem. Cell Biol..

[71] Schlegel N., Burger S., Golenhofen N., Walter U., Drenckhahn D., Waschke J. (2008). The role of VASP in regulation of cAMP- and Rac 1-mediated endothelial barrier stabilization. Am. J. Physiol. Cell Physiol..

[72] Waschke J., Burger S., Curry F.R.E., Drenckhahn D., Adamson R.H. (2006). Activation of Rac-1 and Cdc42 stabilizes the microvascular endothelial barrier. Histochem. Cell Biol..

[73] Gliem M., Heupel W.M., Spindler V., Harms G.S., Waschke J. (2010). Actin reorganization contributes to loss of cell adhesion in pemphigus vulgaris. Am. J. Physiol. Cell Physiol..

[74] Chiariello M., Marinissen M.J., Gutkind J.S. (2001). Regulation of c-myc expression by PDGF through Rho GTPases. Nat. Cell Biol..

[75] Bannai Y., Aminova L.R., Faulkner M.J., Ho M., Wilson B.A. (2012). Rho/ROCK-dependent inhibition of 3T3-L1 adipogenesis by G-protein-deamidating dermonecrotic toxins: Differential regulation of Notch1, Pref1/Dlk1, and β-catenin signaling. Front. Cell. Infect. Microbiol..

[76] Travaglione S., Messina G., Fabbri A., Falzano L., Giammarioli A.M., Grossi M., Rufini S., Fiorentini C. (2005). Cytotoxic necrotizing factor 1 hinders skeletal muscle differentiation in vitro by perturbing the activation/deactivation balance of Rho GTPases. Cell Death Differ..

[77] Kazmierczak B.I., Jou T.S., Mostov K., Engel J.N. (2001). Rho GTPase activity modulates Pseudomonas aeruginosa internalization by epithelial cells. Cell. Microbiol..

[78] Moreau V., Tatin F., Varon C., Génot E. (2003). Actin Can Reorganize into Podosomes in Aortic Endothelial Cells, a Process Controlled by Cdc42 and RhoA. Mol. Cell. Biol..

[79] Baumer Y., Drenckhahn D., Waschke J. (2008). cAMP induced Rac 1-mediated cytoskeletal reorganization in microvascular endothelium. Histochem. Cell Biol..

[80] Vouret-Craviari V., Grall D., Flatau G., Pouysségur J., Boquet P., Van Obberghen-Schilling E. (1999). Effects of cytotoxic necrotizing factor 1 and lethal toxin on actin cytoskeleton and VE-cadherin localization in human endothelial cell monolayers. Infect. Immun..

[81] Travaglione S., Loizzo S., Rizza T., Del Brocco A., Ballan G., Guidotti M., Vona R., Di Nottia M., Torraco A., Carrozzo R. (2014). Enhancement of mitochondrial ATP production by the *Escherichia coli* cytotoxic necrotizing factor 1. FEBS J..

[82] Malorni W., Quaranta M.G., Straface E., Falzano L., Fabbri A., Viora M., Fiorentini C. (2003). The Rac-activating toxin cytotoxic necrotizing factor 1 oversees NK cell-mediated activity by regulating the actin/microtubule interplay. J. Immunol..

[83] Gall-Mas L., Fabbri A., Namini M.R.J., Givskov M., Fiorentini C., Krejsgaard T. (2018). The bacterial toxin CNF1 induces activation and maturation of human monocyte-derived dendritic cells. Int. J. Mol. Sci..

[84] Fabbri A., Travaglione S., Maroccia Z., Guidotti M., Pierri C.L., Primiano G., Servidei S., Loizzo S., Fiorentini C. (2018). The bacterial protein CNF1 as a potential therapeutic strategy against mitochondrial diseases: A pilot study. Int. J. Mol. Sci..

[85] Dufies O., Doye A., Courjon J., Torre C., Michel G., Loubatier C., Jacquel A., Chaintreuil P., Majoor A., Guinamard R.R. (2021). Escherichia coli Rho GTPase-activating toxin CNF1 mediates NLRP3 inflammasome activation via p21-activated kinases-1/2 during bacteraemia in mice. Nat. Microbiol..

[86] Hahn A., Barth H., Kress M., Mertens P.R., Goppelt-Struebe M. (2002). Role of Rac and Cdc42 in lysophosphatidic acid-mediated cyclo-oxygenase-2 gene expression. Biochem. J..

[87] Malchiodi-Albedi F., Paradisi S., Di Nottia M., Simone D., Travaglione S., Falzano L., Guidotti M., Frank C., Cutarelli A., Fabbri A. (2012). CNF1 improves astrocytic ability to support neuronal growth and differentiation in vitro. PLoS ONE.

[88] Boutillier S., Rapp J., Staeb T., Olenik C., Schmidt G., Meyer D.K., Leemhuis J. (2003). Cytotoxic necrotizing factor-2 of Escherichia coli alters the morphology of cultured hippocampal neurons. Naunyn. Schmiedebergs. Arch. Pharmacol..

[89] Musilli M., Ciotti M.T., Pieri M., Martino A., Borrelli S., Dinallo V., Diana G. (2016). Therapeutic effects of the Rho GTPase modulator CNF1 in a model of Parkinson’s disease. Neuropharmacology.

[90] Czopka T., Von Holst A., Schmidt G., Ffrench-Constant C., Faissner A. (2009). Tenascin C and tenascin R similarly prevent the formation of myelin membranes in a RhoA-dependent manner, but antagonistically regulate the expression of myelin basic protein via a separate pathway. Glia.

[91] Fortemaison N., Blancquaert S., Dumont J.E., Maenhaut C., Aktories K., Roger P.P., Dremier S. (2005). Differential involvement of the actin cytoskeleton in differentiation and mitogenesis of thyroid cells: Inactivation of Rho proteins contributes to cyclic adenosine monophosphate-dependent gene expression but prevents mitogenesis. Endocrinology.

[92] Doye A., Boyer L., Mettouchi A., Lemichez E. (2006). Ubiquitin-mediated proteasomal degradation of Rho proteins by the CNF1 toxin. Methods Enzymol..

[93] Haga R.B., Ridley A.J. (2016). Rho GTPases: Regulation and roles in cancer cell biology. Small GTPases.

[94] Ding F., Yin Z., Wang H.-R. (2011). Ubiquitination in Rho Signaling. Curr. Top. Med. Chem..

[95] Yang S., Rosenwald A. (2018). Small GTPase proteins in macroautophagy. Small GTPases.

[96] Fiorentini C., Matarrese P., Straface E., Falzano L., Fabbri A., Donelli G., Cossarizza A., Boquet P., Malorni W. (1998). Toxin-Induced Activation of Rho GTP-Binding Protein Increases Bcl-2 Expression and Influences Mitochondrial Homeostasis. Exp. Cell Res..

[97] Horiguchi Y. (2001). Escherichia coli cytotoxic necrotizing factors and Bordetella dermonecrotic toxin: The dermonecrosis-inducing toxins activating Rho small GTPases. Toxicon.

[98] Chircop M. (2014). Rho GTPases as regulators of mitosis and cytokinesis in mammalian cells. Small GTPases.

[99] El-Aouar Filho R.A., Nicolas A., De Paula Castro T.L., Deplanche M., De Carvalho Azevedo V.A., Goossens P.L., Taieb F., Lina G., Le Loir Y., Berkova N. (2017). Heterogeneous family of cyclomodulins: Smart weapons that allow bacteria to hijack the eukaryotic cell cycle and promote infections. Front. Cell. Infect. Microbiol..

[100] Island M.D., Cui X., Warren J.W. (1999). Effect of Escherichia coli cytotoxic necrotizing factor 1 on repair of human bladder cell monolayers in vitro. Infect. Immun..

[101] Munro P., Flatau G., Lemichez E. (2007). Intranasal immunization with tetanus toxoid and CNF1 as a new mucosal adjuvant protects BALB/c mice against lethal challenge. Vaccine.

[102] Diabate M., Munro P., Garcia E., Jacquel A., Michel G., Obba S., Goncalves D., Luci C., Marchetti S., Demon D. (2015). Escherichia coli α-Hemolysin Counteracts the Anti-Virulence Innate Immune Response Triggered by the Rho GTPase Activating Toxin CNF1 during Bacteremia. PLoS Pathog..

[103] Ahmed A.U., Williams B.R.G., Hannigan G.E. (2015). Transcriptional activation of inflammatory genes: Mechanistic insight into selectivity and diversity. Biomolecules.

[104] Collins P.W., Noble K.E., Reittie J.R., Hoffbrand A.V., Pasi K.J., Yong K.L. (1995). Induction of tissue factor expression in human monocyte/endothelium cocultures. Br. J. Haematol..

[105] Fabbri A., Travaglione S., Fiorentini C. (2010). Escherichia coli cytotoxic necrotizing factor 1 (CNF1): Toxin biology, in vivo applications and therapeutic potential. Toxins.

[106] Omar A., Jovanovic K., Da Costa Dias B., Gonsalves D., Moodley K., Caveney R., Mbazima V., Weiss S.F. (2011). Patented biological approaches for the therapeutic modulation of the 37 kDa/67 kDa laminin receptor. Expert Opin. Ther. Pat..

[107] Gopalakrishna R., Bhat N.R., Zhou S., Mack W.J. (2019). Cell signaling associated with internalization of 67 kDa laminin receptor (67LR) by soluble laminin and its implication for protection against neurodegenerative diseases. Neural Regen. Res..

[108] Loizzo S., Rimondini R., Travaglione S., Fabbri A., Guidotti M., Ferri A., Campana G., Fiorentini C. (2013). CNF1 Increases Brain Energy Level, Counteracts Neuroinflammatory Markers and Rescues Cognitive Deficits in a Murine Model of Alzheimer’s Disease. PLoS ONE.

[109] Cloutier J.M., Charville G.W. (2019). Diagnostic classification of soft tissue malignancies: A review and update from a surgical pathology perspective. Curr. Probl. Cancer.

[110] Kashina A.S. (2020). Regulation of actin isoforms in cellular and developmental processes. Semin. Cell Dev. Biol..

[111] Travaglione S., Fabbri A., Fiorentini C. (2008). The Rho-activating CNF1 toxin from pathogenic E. coli: A risk factor for human cancer development?. Infect. Agent. Cancer.

[112] Fabbri A., Travaglione S., Ballan G., Loizzo S., Fiorentini C., Fabbri A., Travaglione S., Ballan G., Loizzo S., Fiorentini C. (2013). The Cytotoxic Necrotizing Factor 1 from E. Coli: A Janus Toxin Playing with Cancer Regulators. Toxins.

[113] Piciocchi A., Germinario E.A.P., Garcia Etxebarria K., Rossi S., Sanchez-Mete L., Porowska B., Stigliano V., Trentino P., Oddi A., Accarpio F. (2021). Association of polygenic risk score and bacterial toxins at screening colonoscopy with colorectal cancer progression: A multicenter case-control study. Toxins.

