# Supplementary figures and images for: Effects of the Escherichia coli Bacterial Toxin Cytotoxic Necrotizing Factor 1 on Different Human and Animal Cells: A Systematic Review
Source: Int J Mol Sci. 2021 Nov 22;22(22):12610. doi: 10.3390/ijms222212610 (PMC8621085; doi:10.3390/ijms222212610)

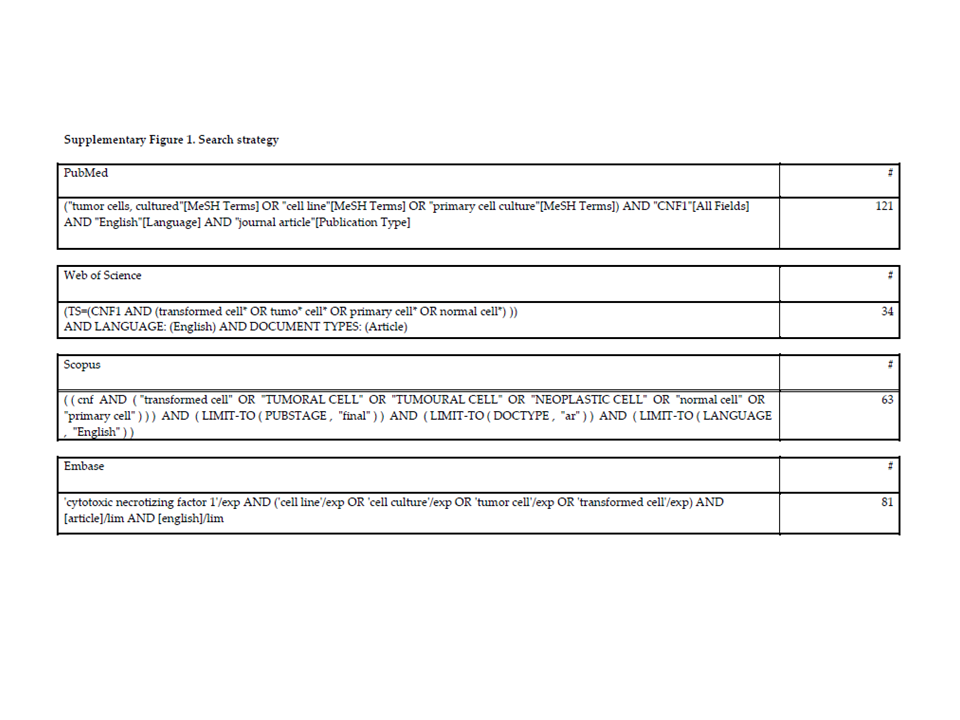

Supplement: Supplementary file 1 [file ijms-22-12610-s001.zip › ijms-1457623-supplementary.tif]
